# Empagliflozin Protects against Haloperidol Experimentally-Induced Ovarian Toxicity

**DOI:** 10.3390/ph16020168

**Published:** 2023-01-23

**Authors:** Walaa Yehia Abdelzaher, Michel De Waard, Alyaa Abdelfattah Abdelmonaem, Dalia Mohamed Ali, Nashwa Fathy Gamal El-Tahawy, Rehab Ahmed Rifaai, Hatem A. Mohamed, Kareem Shaheen, Mohamed Ahmed Zeen El-Din, Nermeen N. Welson, Shereen ELsayed Tawfeek, Gaber El-Saber Batiha, Asmaa Mohamed Abdel-Aziz

**Affiliations:** 1 Department of Pharmacology, Faculty of Medicine, Minia University, Minia 61511, Egypt; 2 L’institut Du Thorax, INSERM, CNRS, UNIV NANTES, F-44007 Nantes, France; 3 Smartox Biotechnology, 6 Rue des Platanes, F-38120 Saint-Egrève, France; 4 LabEx «Ion Channels, Science & Therapeutics», Université de Nice Sophia-Antipolis, F-06560 Valbonne, France; 5 Department of Forensic Medicine and Clinical Toxicology, Faculty of Medicine, Minia University, Minia 61511, Egypt; 6 Department of Histology and Cell Biology, Faculty of Medicine, Minia University, Minia 61511, Egypt; 7 Department of Biochemistry, Faculty of Medicine, Minia University, Minia 61511, Egypt; 8 Department of Obstetrics & Gynecology, Faculty of Medicine, Minia University, Minia 61511, Egypt; 9 Department of Forensic Medicine and Clinical Toxicology, Faculty of Medicine, Beni-Suef University, Beni Suef 62511, Egypt; 10 Anatomy Department, College of Medicine, Jouf University, Sakaka 72388, Saudi Arabia; 11 Human Anatomy and Embryology Department, Faculty of Medicine, Zagazig University, Sharkia 44519, Egypt; 12 Department of Pharmacology and Therapeutics, Faculty of Veterinary Medicine, Damanhour University, Damanhour 22511, Egypt

**Keywords:** empagliflozin, haloperidol, ovarian tissue, Hsp70, Sirt-1, caspase-3

## Abstract

The present experiment aimed to identify the potential protective role of empagliflozin (EMPA) on haloperidol (HAL)-induced ovarian damage in female rats because of its anti-inflammatory, antioxidant, and antiapoptotic effects. EMPA was administered in the presence and absence of HAL. Thirty-two adult female albino rats were divided into four groups. Control group, EMPA group: received EMPA (10 mg/kg/day) p.o., HAL group: received HAL (2 mg/kg/day) p.o., HAL + EMPA group: HAL (2 mg/kg/day) combined with EMPA for 28 days. Serum follicle-stimulating hormone (FSH), luteinizing hormone (LH), and anti-mullerian hormone (AMH) levels were measured. Ovarian oxidative stress parameters, besides inflammatory and apoptotic biomarkers, and ovarian Sirtuin-1 (Sirt-1) were evaluated. Ovarian histopathological examination and heat shock protein 70 (Hsp70) immunohistochemical study were performed. HAL significantly increased serum levels of FSH, LH, and ovarian inflammatory, apoptotic, and oxidative stress biomarkers and decreased serum AMH levels and Sirt-1 expression. Histopathological findings of ovarian damage and high Hsp70 immunoexpression were detected. EMPA significantly normalized the distributed hormonal levels, oxidative stress, inflammatory, and apoptotic biomarkers with a prompt improvement in the histopathological picture and a decrease in Hsp70 immunoexpression. Accordingly, EMPA protected against HAL-induced ovarian toxicity by modulating the Sirt-1/Hsp70/TNF-α/caspase-3 signaling pathway.

## 1. Introduction

Psychiatric diseases are major issues that reduce people’s quality of life. They are more frequently seen in women, nearly two times more than in men. One of them is schizophrenia, which is a severe chronic disorder affecting 20 million people worldwide and occurs in repetitive attacks. Long-term management is required, even though it can be lifelong, affecting the lives of the patients and their families [1,2]. Unfortunately, antipsychotic agents such as haloperidol (HAL) produce a lot of side effects, particularly in female patients, disturbing their reproductive ability, as the female reproductive system is very sensitive to different harmful factors [3].

Chronic HAL treatment can cause reproductive dysfunction via a variety of mechanisms, including oxidative stress in mitochondrial ovarian follicles, resulting in premature ovarian failure (POF) and ovarian function cessation [4], and also via its effect on the hypothalamo–hypophyseal ovarian axis [5].

Reactive Oxygen Species (ROS) production impairs ovarian antioxidant defenses, as well as lipid peroxidation and mitochondrial injury, resulting in caspase activation and ovarian follicle apoptosiss [6].

There has been increasing concern about the maintaining and restoration of fertility before and during psychiatric therapy, considering its reproductive complications. Basal measurements of follicle-stimulating hormone (FSH), luteinizing hormone (LH), and antimullerian hormone (AMH), as direct indicators of the follicular pool and ovarian imaging, are used as ovarian reserve tests to predict reproductive function [7].

Nuclear factor (erythroid-derived 2)-like 2 (Nrf2) has a vital role in the antioxidant and anti-inflammatory responses with a cytoprotective effect on ovarian tissue via regulating the basal and inducible expression of antioxidant proteins, detoxification enzymes, and xenobiotic transporters [8]. On the other hand, silent information regulator 1, or Sirtuin type 1 (Sirt1), a NAD-dependent histone deacetylase, is highly expressed in ovarian tissue, which stimulates an antioxidant effect that restores cells hurted by oxidative damage. Its decrease leads to mitochondrial dysfunction and infertility. Thus, because of increasing ROS, lipid peroxidation, and DNA damage in both male and female gametes, its down-regulation is linked to a reduced ovarian reserve [9].

Unlike conventional drugs, empagliflozin (EMPA) is an antidiabetic drug that acts via the suppression of sodium-glucose co-transporter-2 (SGLT-2) that is abundantly present in both rats and humans [10]. EMPA could exhibit antioxidant and anti-inflammatory effects via a SGLT-2-independent pathway, activating the intracellular nuclear factors; erythroid 2–related factor 2 (Nrf2) and sirtuin 1 (Sirt1). Thus, the expression of various antioxidants, as well as the suppression of oxidative stress and the reduction of superoxide synthesis, suppress subsequent inflammatory cytokine production [11,12].

Given previous reports on the function of EMPA, the present experiment was designed to look into the possible role of EMPA in mitigating oxidative stress, inflammation, and apoptosis in adult female albino rats, as well as the possible mechanisms underlying its action, with a focus on the Sirt-1/Hsp70/TNF-α/caspase-3 signaling cascade.

## 2. Results

### 2.1. Effect of EMPA on Oxidative Stress Parameters in HAL-Induced Ovarian Toxicity

The HAL group showed a significant elevation in ovarian MDA and NOx with a significant inhibition in ovarian SOD when compared to the control and EMPA groups. On the other hand, rats co-treated with HAL + EMPA had a significant improvement in the aforementioned parameters in comparison to the HAL group (Table 1).

### 2.2. Effect of EMPA on Hormonal Parameters in HAL-Induced Ovarian Toxicity

The HAL group significantly increased levels of FSH and LH and decreased the level of AMH when compared to the control and EMPA groups. A significant decrease in levels of FSH and LH and a significant increase in the level of AMH were demonstrated in the HAL + EMPA group in comparison to the HAL group (Table 2).

### 2.3. Effect of EMPA on Inflammatory and Apoptotic Parameters in HAL-Induced Ovarian Toxicity 

In comparison to the control and EMPA groups, the HAL group had a significant increase in ovarian TNF-α, IL-6, and caspase-3 and a significant decrease in ovarian Nrf2. Meanwhile, when compared to the HAL group, the HAL + EMPA group showed a significant decrease in ovarian TNF-α, IL-6, and caspase-3 with a significant increase in ovarian Nrf2 (Table 3).

### 2.4. Effect of EMPA on Expression of Sirt-1 in HAL-Induced Ovarian Toxicity in Rats

**Figure 1:**
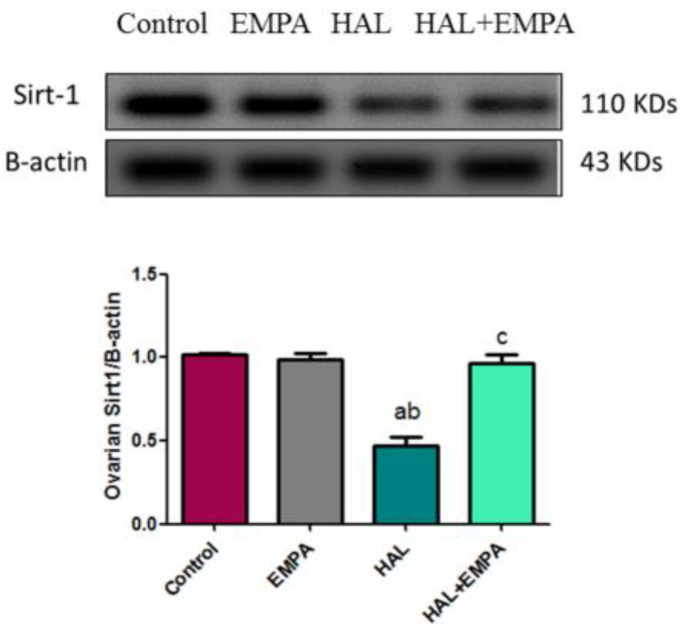
Effect of EMPA on Sirt-1 Western blotting in HAL-induced ovarian toxicity in rats. Results represent the mean ± SEM (8 rats/group). ^a^ Significant (*p* < 0.05) difference from the control group. ^b^ Significant (*p* < 0.05) difference from the EMPA group. ^c^ Significant (*p* < 0.05) difference from HAL. [EMPA = empagliflozin; HAL = haloperidol; Sirt-1= Sirtuin 1].

The HAL group showed a significant decrease in ovarian Sirt-1 expression in comparison to the control and EMPA groups. The HAL + EMPA group showed a significant increase in ovarian Sirt-1 expression when compared to the HAL group (Figure 1).

### 2.5. Histological Results

**Figure 2:**
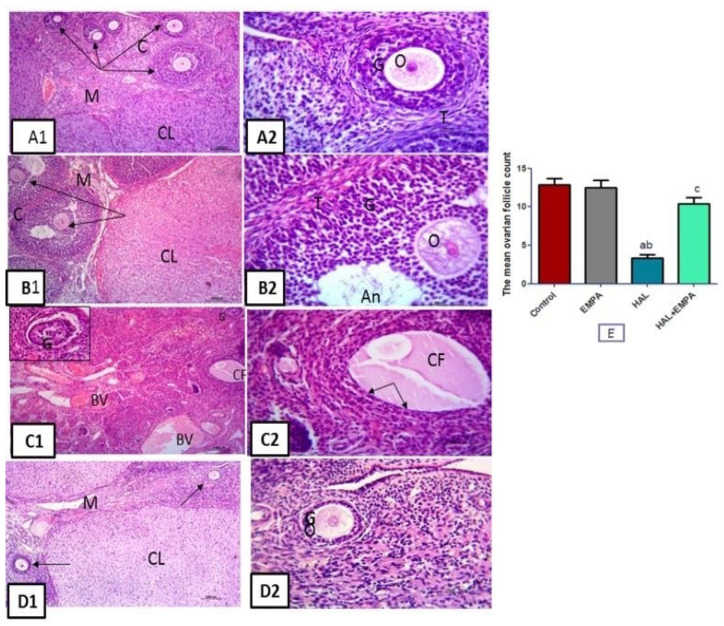
Photomicrographs of sections in the ovary: (**A1**) control and (**B1**) EMPA groups, respectively, showed normal ovarian structure with inner vascularized medulla (M) and outer cortex (C) with numerous growing follicles formed of oocytes with the surrounding granulosa cells (arrows) and corpus luteum (CL). (**A2**,**B2**) are higher magnification for the growing follicles from control and EMPA groups, respectively showing oocytes with prominent nucleus (O) surrounded by layers of granulosa cells (G) and outer theca cells (T). Notice the follicular antrum (An). (**C1**,**C2**) of HAL group showed the ovarian medulla with marked congested blood vessels (BV). The cortex appears with hardly seen follicles. Large cystic follicle (CF) and degenerated follicle with detached degenerated granulosa cells (G) (inset) are noticed. The cystic follicle lined by thin attenuated flat granulosa cells (arrows) with absent oocyte. (**D1**,**D2**) of HAL + EMPA group showed ovarian structure appears more or less normal with normal medulla (M) and cortex (C) with many growing follicles (GF) (arrows) and corpus luteum (CL). (**D2**) is higher magnification for a growing follicle showing normal oocytes(O) surrounded by granulosa cells (G) H&E; (**A1**,**B1**,**C1**,**D1**) X100 scale bar 100 µ (**A2**,**B2**,**C2**,**D2**) X100 scale bar 50µ. (**E**) The mean number of follicles in the studied groups expressed as mean ± S.E.M (n = 8). ^a^ is significantly different from control, ^b^ is significantly different from EMPA and ^c^ is significantly different from HAL. (*p* < 0.05) using one-way ANOVA followed by Tukey–Kramer post hoc test.

The ovarian tissues of the control and EMPA groups showed normal structure with an inner vascularized medulla and an outer cortex occupied with numerous growing follicles of different sizes and stages and many corpora lutea. Each growing follicle showed normal oocytes with a prominent nucleus, surrounded by layers of granulosa cells and theca cells. Tissues from the HAL group showed marked structural changes. The blood vessels of the ovarian medulla appeared markedly congested. Scanty follicles were noticed within the cortex. The cortical follicles were replaced either by cystic follicles, which showed absent oocytes and were lined by thin, attenuated flat granulosa cells, or by degenerated follicles with detached degenerated granulosa cells. Regarding ovarian tissue from the HAL + EMPA group, more or less normal structure was observed with normal medulla and cortex. The cortex was occupied by many growing follicles with normal oocytes, granulosa cells, and stromal theca cells. The mean ovarian follicle count showed a significant decrease in the HAL group when compared to the control and EMPA groups The HAL + EMPA group showed a significant increase in the mean ovarian follicle count when compared to the HAL group (Figure 2).

### 2.6. Immunohistochemical Results

**Figure 3:**
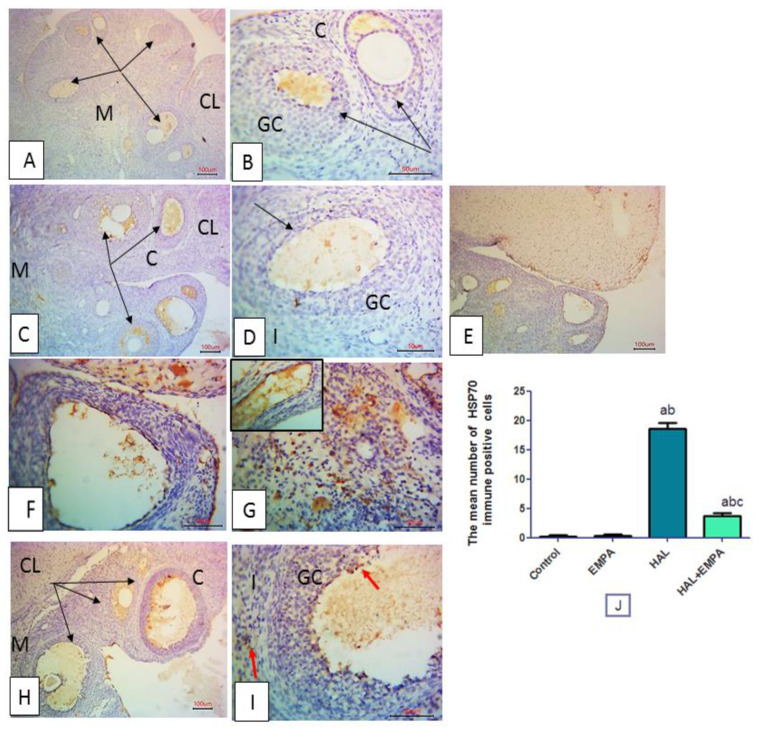
Photomicrographs of rat ovarian tissues immunostained for HSP70. The control group (**A**,**B**) and group 2 (**C**,**D**) showing negative immune staining, group-3 (**E**–**G**) showing HSP70 positive immune-expression (red arrows) either in the granulosa cells, interstitial cells and endothelial lining of blood vessel blood vessels. Group 4 (**H**) showing scarcely immune-positive cells (red arrows) of the granulosa and interstitial cells. Growing follicles at different stages (black arrows); corpus luteum (CL), granulosa cells (GC), interstitial cells (**I**), cystic follicle (CF) and blood vessels (BV). Immunohistochemistry, counterstained with H; (**A**,**C**,**E**,**H**) × 100 scale bar 100µ and (**B**,**D**,**F**,**G**,**I**) × 400. (**J**) The mean number of Hsp70 immune-positive cells in the studied groups expressed as mean ± S.E.M (n = 8). ^a^ is significantly different from control, ^b^ is significantly different from EMPA group and ^c^ is significantly different from HAL group at *p* < 0.05 using one-way ANOVA followed by Tukey–Kramer post hoc test.

Immune-stained ovarian tissues for Hsp70 from the control and EMPA groups displayed negative immune expression. The HAL group showed significantly increased positive immune expression in the granulosa cells, interstitial cells, and endothelial lining of blood vessels. While the HAL + EMPA-treated group showed significantly diminished immune reactivity compared to the HAL group (Figure 3).

## 3. Discussion

Since the 1950s, antipsychotic medications have been mostly prescribed for psychosis and bipolar disorders. Typical antipsychotics such as HAL produce multiple side effects such as extrapyramidal manifestations, weight gain, and reproductive disorders in young females [13].

Different mechanisms were suggested to explain HAL-induced ovarian toxicity, such as ovarian oxidative stress, inflammation, and apoptosis. ROS and oxidative stress have been shown to have an effect on young female reproductive capacity [14]. Oxidative stress occurred if the formation of ROS exceeded the ability of the ovarian cells to defend themselves from ROS by having sufficient levels of antioxidant enzymes. Antioxidants are critical for preserving ovarian tissue function [4].

The disparity between free radical production and the antioxidant defense system and the resultant oxidative stress was proven with HAL administration in the current study, as the ovarian antioxidant enzymes, SOD and Nrf2, were significantly decreased with the lowered expression of ovarian Sirt-1. On the other hand, increased ROS levels in the form of ovarian MDA occurred. MDA is a signal of oxidative stress and the last step of lipid peroxidation. These results are in line with Elmorsy and Smith [15]. Apoptosis of granulosa cells and ovarian follicle atresia resulted from oxidative stress during HAL-toxicity induction [16,17]. In the present study, the loss of follicles after HAL administration was confirmed by the histopathological examination and caspase-3 ovarian tissue level, thus reflecting the oxidative stress-induced follicular atresia with HAL.

The current study found a decrease in serum AMH levels as well as increases in serum FSH and LH levels, which increased follicular atresia and decreased the follicular number due to granulosa cell apoptosis and proliferation inhibition, as previously suggested [18]. Altered negative feedback signals may be the main cause of disturbed levels of FSH and LH [19]. 

ROS and reactive nitrogen species production are caused by the reaction of NO with the superoxide radical (O2). Therefore, multiple diseases resulting from oxidative stress and inflammatory conditions were caused by NO, which damages cells wherever it is produced [20]. In this study, a significant increase in NOx levels was present after HAL use, indicating its paramount role in the pathogenesis of HAL-induced ovarian injury. Overabundant NOx levels could inhibit progesterone production and produce apoptotic cell death in rat granulose cells [21].

Heat-shock proteins (HSPs) act as intracellular chaperones that protect cells from abnormally folded or mutated proteins, which may be involved in different stressful conditions. HSP upregulation occurs as a result of several stresses, including inflammation. Hsp70, in particular, has a cytoprotective role in vitro and in vivo via its anti-apoptosis processes [21]. Hsp70 inhibits proinflammatory cytokine formation in a variety of cells with a reduction in NF-kB activation and its downstream TNF-α and IL-6 levels [22]. The current study reported that HAL significantly increased the immunoexpression of ovarian Hsp70. Hsp70 was also found to control local tissue inflammation by suppressing the intracellular apoptotic cascade and preventing the irreversible aggregation of heat-damaged protein [23].

In the current study, HAL caused a significant increase in TNF-α and IL-6 ovarian levels, indicating an inflammatory role on ovarian tissue that can activate the pro-apoptotic caspase cascade, leading to ovarian follicle apoptosis [24]. Anti-inflammatory and antioxidant medications would be useful in the prevention of HAL-induced ovarian toxicity, according to these findings.

EMPA is an antidiabetic drug that inhibits SGLT2, which offers clinically meaningful protection in type 2 diabetic individuals [25]. Attenuation of oxidative stress, inflammation, and apoptosis are considered some of the postulated mechanisms of EMPA effects in tissue protection [26,27].

In this regard, the current study aimed to investigate the potential efficacy of EMPA treatment against HAL-induced ovarian injury in rats and show the underlying mechanisms that mediate its possible ameliorative effect.

Present results revealed that the EMPA-treated group in HAL-induced ovarian toxicity had significant improvements in all parameters related to ovarian oxidative stress, inflammation, and apoptosis, with restoration of the normal ovarian histopathological picture besides gonadotropins’ hormonal balance, when compared to HAL-non-treated rats, and these results are consistent with other previous studies [28,29]. Several studies have detected the expression of SGLT in mammalian and rat follicles, showing their importance in oocyte maturation and ovarian cellular glucose metabolism [30].

As EMPA exhibits a unique antioxidant activity [29,31], in the current study, EMPA showed an eminent antioxidant potential via its ability to compensate the oxidative damage caused by HAL, as demonstrated by elevated ovarian tissue SOD and Nrf2 levels along with Sirt-1 expression in the ovarian tissue, as well as reductions in MDA and NOx ovarian contents, owing to its free radical scavenging ability.

TNF-α and IL-6 cytokines produced by immune cells [32] have a prominent role in the pathogenesis of HAL-induced ovarian toxicity [24], varying from inflammatory progression up to end-organ damage [33], as TNF-α stimulates various apoptotic proteins, activating the apoptotic degradation phase [34]. In the current study, EMPA significantly reduced ovarian Hsp70 immunoexpression as well as ovarian TNF-α and IL-6 levels. Our results are in line with many previous studies [35,36].

With the suppression of the inflammatory cascade, EMPA pretreatment successfully decreased the ovarian caspase-3 level, as consistent with other previous studies that concluded the ability of EMPA to reduce caspase-3 expression in rats with diabetic cardiomyopathy as caspase-3 is an important cell marker for the apoptotic signal pathway [36,37].

Interestingly, EMPA treatment reduced the level of the apoptotic marker caspase 3, as well as having significant anti-inflammatory and antioxidant effects. The anti-inflammatory and antioxidant effects of it may explain the ameliorations in declining ovarian functions and the histopathological changes induced by HAL in the form of follicular number preservation and the decrease in follicular atresia, as well as the increase in serum AMH level and the decrease in serum FSH and LH levels.

Another possible explanation of EMPA-mediated improvement of ovarian functions in HAL-induced ovarian toxicity is the abrogation of HAL-induced decline in ovarian sirtuin-1 levels in the EMPA-treated group. Sirtuin-1, a member of the seven mammalian sirtuins, is responsible for histone deacetylation and transcriptional regulation. Increased Sirt-1 expression was linked to increased expression of antioxidant enzymes such as SOD in this study and others. Further, Sirt-1 exhibits antiapoptotic and antifibrotic effects. It has a role in the improvement of recovery after tissue injury [38]. We hypothesised that HAL-induced ovarian injury may be mediated via the decline in ovarian Sirt1. In the present result, ovarian Sirt1 gene expression decreased in the HAL group, which was parallel to the observed decrease in ovarian functions and antioxidant activity together with the increase in ovarian inflammation and apoptosis. These effects were ameliorated via treatment with EMPA, suggesting a possible role for Sirt1 in EMPA-mediated ovarian protective effects.

## 4. Materials and Methods

### 4.1. Animals and Experimental Design

Thirty-two adult female albino rats, aged 3–4 months and weighing about 210–230 g, were included in the present work. Animals were obtained from the Animal Research Center, Giza, Egypt. Rats were housed in a standard housing state (3 rats per cage), had free chow and tap water access, and were left for one week for acclimatization. The study was approved by the ethical committee of the Faculty of Medicine, Minia University, Egypt, according to the NIH Guide for the Use of Laboratory Animals in Research (Approval No. 391:2022). Four groups of eight rats each were randomly assigned:

(1) Control group: only the vehicle was given.
(2) EMPA group: received EMPA (10 mg/kg/day) p.o. dissolved in distilled water for 28 days [39,40].
(3) HAL group: received HAL (2 mg/kg/day) p.o. dissolved in distilled water for 28 days [4,41].
(4) HAL + EMPA group: HAL (2 mg/kg/day) PO for 28 days was combined with EMPA (10 mg/kg/day) PO for 28 days.

### 4.2. Chemicals and Drugs

EMPA was obtained from Boehringer Ingelheim (Ingelheim, Germany). HAL was obtained from Kahira Pharmaceuticals (Cairo, Egypt). Anti-mullerian hormone (AMH) (Catalog No: MBS701712), follicle-stimulating hormone (FSH) (Catalog No: MBS2502190), luteinizing hormone (LH) (Catalog No: MBS764675), Nrf2 (Catalog No: MBS752046) and caspase-3 (Catalog No: MBS261814) were measured by ELISA kits (MyBioSource, San Diego, USA). TNF-α (Catalog No: BMS622), IL-6 (Catalog No: ERA31RB) ELISA kits were obtained from Thermo Fisher Scientific Inc., Waltham, MA, USA). Rabbit anti-beta-actin antibody (1:1000, Catalog No: ab8227, Abcam, Cambridge, UK) and Rabbit anti-Sirt1 antibody (1:1000, Catalog No: ab189494, Abcam) were applied.

### 4.3. Blood and Tissue Samples

At the termination of the experiment, IP injections of urethane (25% in a dose of 1.6 g/kg) were used to anesthetize the rats before scarification. Blood samples were taken from the abdominal aorta using heparinized syringes and centrifuged at 4000× *g* for 15 min (Janetzki T30 centrifuge, Germany). Sera were collected and stored at 80 °C for use in biochemical studies. After rinsing with saline and removing attached adipose tissue, the ovaries were divided into two parts. The first part was for the biochemical investigations, and the second one was put in 10% formalin for the histological assessment.

### 4.4. Biochemical Determination

#### 4.4.1. Estimation of the Oxidative Stress Indicators

The left ovaries of each group were homogenized in potassium phosphate buffer (10 mM, pH 7.4) with a ratio of 1 tissue: 5 homogenization buffers. After centrifuging at 4000× *g* for 10 min at 4 °C, the supernatant was used for measuring malondialdehyde (MDA), superoxide dismutase (SOD), and total nitrite/nitrate (NOx).

As an indicator of lipid peroxidation, MDA was measured in accordance with the method described by Buege and Aust, 1978 [42]. 

The method of Marklund and Marklund, 1974 [43], with a little modification, was used to measure the activity of SOD.

Because NOx is a stable end product of nitric oxide oxidation, it was used as a nitric oxide level indicator. It was detected by reducing nitrate into nitrite with activated cadmium granules, and color formation with the Griess reagent in an acidic medium [44]. 

#### 4.4.2. Estimation of Hormonal Parameters

Serum AMH, FSH, and LH were evaluated with their specific ELISA kits under the guidance of the manufacturer’s instructions.

#### 4.4.3. Estimation of Inflammatory and Apoptotic Parameters

The levels of Nrf2, IL-6, TNF-α, and caspase-3 in ovarian tissue were measured using their specific ELISA kits.

### 4.5. Immunoblotting of Sirt1

Ovarian homogenates (50 g total proteins) were heated for five minutes in a 2-mercaptoethanol-containing loading buffer before being run for two hours at 100 V on a 12% sodium dodecyl sulfate-polyacrylamide gel electrophoresis (SDS-PAGE). Proteins were electrophoretically separated and applied to polyvinylidene fluoride (PVDF) membranes. Blocking was performed for one hour in a Tris-buffered saline (TBS-T) solution containing 5% (*w*/*v*) nonfat milk and 0.05% Tween-20. Rabbit anti-Sirt1 and *B*-actin primary antibodies (1:1000) were incubated overnight at 4 °C. In blocking buffer, a secondary antibody of horseradish peroxidase-conjugated polyclonal goat anti-rabbit immunoglobulin was added at a dilution of 1:5000. Chemiluminescence was used to visualize bands using a luminescent image analyzer and chemiluminescence kits (LAS-4100, Fujifilm Co., Tokyo, Japan). After standardization to *B*-actin, protein bands from various groups were analyzed as fold changes compared to the sham group using Image J software.

### 4.6. Histological Study

#### 4.6.1. Histological Procedures 

Specimens of the rat ovaries were obtained, then fixed in 10% neutral formaldehyde, washed, dehydrated by alcohol, cleared by xylol, and finally embedded in paraffin. 5 μm thick, the obtained sections were stained with hematoxylin (H) and eosin (E) [45].

#### 4.6.2. Immunohistochemical Study

Five μm paraffin sections were treated with H_2_O_2_, washed in tris buffer saline, and incubated with rabbit anti-rat Hsp70 antibody (Heat shock protein 70, ab181606, Abcam, UK), a molecular chaperone that is expressed in response to stress. Nonspecific protein binding sites were blocked with normal goat anti-rabbit antibody (1 h), followed by the Avidin-Biotin Complex (1 h), DAB (10 min), and finally hematoxylin counterstained sections [46].

#### 4.6.3. Image Capture

Sections stained with H&E and immunostained for Hsp70 were examined and analyzed using a light microscope (Olympus, Tokyo, Japan). Images were digitally taken by a ToupView camera (ToupView, Zhejiang, China), which was controlled by the Toup View software (version ×36, 3.5.563; Hangzhou Toup Tek Photonics Co., Zhejiang, China).

#### 4.6.4. Morphometrical Measurement

The mean ovarian follicle (all types of follicles) count was measured on the H&E-stained slides.

Hsp70-Immune-stained sections: the mean number of HSP70 immune-positive cells in three non-overlapping fields per section was counted.

### 4.7. Statistical Analysis

One-way ANOVA followed by Tukey’s multiple comparison test were used. The results were presented as means SEM. GraphPad Prism software (version 5) was used for analysis. A *p*-value < 0.05 was set for significance.

## 5. Conclusions

Our findings confirmed that EMPA had beneficial effects in the female rat model of HAL-induced ovarian toxicity. The EMPA treatment reduced elevated levels of FSH and LH while increasing low levels of AMH. In addition, it demonstrated strong antioxidant properties in the ovaries. Furthermore, EMPA displayed effective anti-inflammatory activities by aborting TNF-α and IL-6 and suppressing Hsp70mmunoexpression and caspase-3 level. The findings established Sirt-1/Hsp70/TNF-α /caspase-3 as the underlying signalling pathway for EMPA alleviation. Taken together, EMPA can mitigate HAL-induced ovarian toxicity via its antioxidant, anti-inflammatory, and anti-apoptotic activities, suggesting its therapeutic potential to control reproductive and metabolic disorders in patients with HAL ovarian injury.

## Figures and Tables

**Table 1:** Effect of EMPA on oxidative stress parameters in HAL-induced ovarian toxicity in rats.

Group	Ovarian MDA (nmol/g Tissue)	Ovarian SOD (U/g Tissue)	Ovarian NOx (nmol/g Tissue)
Control	13.41± 0.91	256.00 ± 6.38	52.34 ± 2.28
EMPA	13.10 ± 0.90	257.50 ± 6.52	53.88 ± 2.74
HAL	50.92 ± 3.17 ^ab^	95.78 ± 4.67 ^ab^	127.20 ± 3.15 ^ab^
HAL + EMPA	18.71 ± 1.65 ^c^	241.7 ± 4.54 ^c^	64.77 ± 3.32 ^ac^

Results represent the mean ± SEM (8 rats/group). ^a^ Significant (*p* < 0.05) difference from the control group. ^b^ Significant (*p* < 0.05) difference from the EMPA group. ^c^ Significant (*p* < 0.05) difference from HAL. [EMPA = empagliflozin; HAL = haloperidol; MDA = malondialdehyde; SOD = superoxide dismutase and NOx = total nitrite/nitrate].

**Table 2:** Effect of EMPA on hormonal parameters in HAL-induced ovarian toxicity in rats.

Group	FSH (IU/L)	LH (IU/L)	AMH (ng/mL)
Control	7.68 ± 0.62	5.52 ± 0.42	7.84 ± 0.59
EMPA	7.16 ± 0.61	7.41 ± 0.67	7.70 ± 0.69
HAL	15.48 ± 1.14 ^ab^	16.89 ± 1.54 ^ab^	2.17 ± 0.15 ^ab^
HAL + EMPA	8.344 ± 0.75 ^c^	8.78 ± 0.58 ^c^	6.70 ± 0.59 ^c^

Results represent the mean ± SEM (8 rats/group). ^a^ Significant (*p* < 0.05) difference from the control group. ^b^ Significant (*p*< 0.05) difference from the EMPA group. ^c^ Significant (*p* < 0.05) difference from HAL. [EMPA = empagliflozin; HAL = haloperidol; FSH = follicle-stimulating hormone; LH = luteinizing hormone; AMH = Anti-mullerian hormone].

**Table 3:** Effect of EMPA on inflammatory and apoptotic parameters in HAL-induced ovarian toxicity in rats.

Group	Ovarian Nrf2 (pg/g Tissue)	Ovarian TNF-α (pg/g Tissue)	Ovarian IL-6 (pg/g Tissue)	Ovarian Caspase-3 (ng/g Tissue)
Control	87.60 ± 4.94	86.17 ± 5.88	97.75 ± 4.24	8.845 ± 0.64
EMPA	86.05 ± 4.37	85.10 ± 7.70	104.9 ± 4.17	9.27 ± 0.66
HAL	46.75 ± 4.22 ^ab^	180.00 ± 6.90 ^ab^	182.40 ± 6.57 ^ab^	23.35 ± 1.57 ^ab^
HAL + EMPA	81.49 ± 6.60 ^c^	94.43 ± 8.50 ^c^	107.90 ± 2.98 ^c^	10.19 ± 0.60 ^c^

Results represent the mean ± SEM (8 rats/group). ^a^ Significant (*p* < 0.05) difference from the control group. ^b^ Significant (*p* < 0.05) difference from the EMPA group. ^c^ Significant (*p* < 0.05) difference from HAL. [EMPA = empagliflozin; HAL = haloperidol; Nrf2 = nuclear factor erythroid 2–related factor 2; TNF-α = tumor necrosis factor alpha; IL-6 = interlukin-6].

## Data Availability

All data are fully available and included in the manuscript.
